# Electronic Cigarette Use (Vaping) Among Adolescents: A Narrative Review of an Emerging Public Health Epidemic

**DOI:** 10.7759/cureus.89422

**Published:** 2025-08-05

**Authors:** Arshia Khodaee, Alex Reed, Morteza Khodaee

**Affiliations:** 1 Biology, University of Wisconsin-Madison, Madison, USA; 2 Family Medicine, University of Colorado School of Medicine, Denver, USA; 3 Sports Medicine, University of Colorado School of Medicine, Denver, USA

**Keywords:** addiction, behavioral health, nicotine, tobacco, vaping devices

## Abstract

The current electronic cigarette (e-cigarette) format was made known to the public in recent decades. Since then, it has gained widespread popularity, particularly among adolescents. A significant portion of young people in the United States and around the world are reported to use e-cigarettes. The reasons for its popularity might be related to its many enticing flavors and being rechargeable. There are many different delivery systems and devices used for e-cigarettes. It took some time for governing organizations, such as the United States Food and Drug Administration (FDA), to place limitations and regulations on the distribution, advertisement, and easy access by minors. E-cigarettes have been advertised as a safer version of the typical (combustible) cigarettes. Nicotine is the main ingredient of most e-cigarettes. Small amounts of other chemicals, including heavy metals and known carcinogens, have also been detected. Evidence of the harmful nature of e-cigarettes is emerging and alarming. Short-term health effects of e-cigarette use may include oral, respiratory, and cardiac illnesses. Long-term outcomes of e-cigarette use, particularly among adolescents, are unknown. E-cigarettes may help with smoking cessation in adults, but their benefits among adolescents are lacking. The purpose of this narrative review is to summarize the risk factors and discuss potential complications associated with adolescent e-cigarette usage. We will also discuss the evidence for concurrent use of other legal and illegal substances with e-cigarettes. Evidence for the most effective ways for cessation is lacking, and further research is needed.

## Introduction and background

The global nicotine market has undergone significant changes with the emergence of products marketed as safer alternatives to traditional combustible cigarettes [1-3]. Among these are heated tobacco products (HTPs) and e-cigarettes, both smoke-free nicotine delivery systems that differ in design and function [1-3]. E-cigarettes work by heating a liquid, usually containing nicotine and flavorings, to produce an inhalable aerosol [1,2,4]. In contrast, HTPs heat specially processed tobacco sticks without burning them [3].

**Figure 1:**
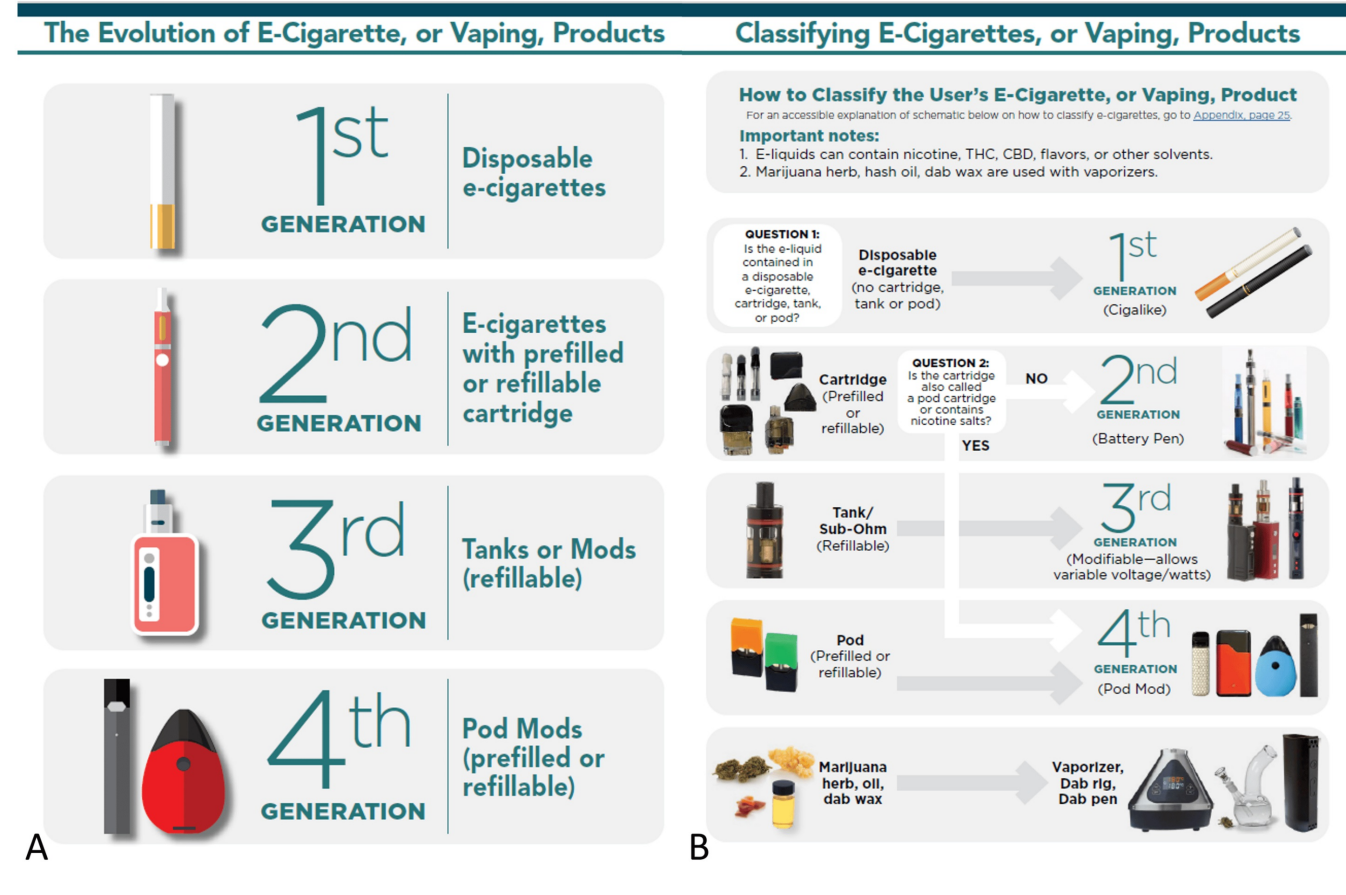
The evolution (A) and classification (B) of e-cigarettes, vaping, and their products. Source: US Centers for Disease Control and Prevention (public domain) [4].

Over the past two decades, e-cigarette use has increased dramatically, emerging as a major public health concern [5-8]. Originally introduced as closed, single-use devices, e-cigarettes have evolved into sophisticated, open-system, rechargeable products that allow users to customize features such as flavor, heating mechanisms, nicotine delivery, and puff volume (Figure 1) [1,2,4,9-11].

Currently, around 10% of adolescents in the United States and globally report using e-cigarettes [12]. Their popularity among youth is driven by several factors, including a wide variety of appealing flavors, easy access, user-friendly design, high susceptibility to nicotine addiction during adolescence, and strong social influences [2,9-18]. In addition, many young users perceive e-cigarettes as a safer alternative to traditional combustible cigarettes, further contributing to their widespread use [10].

**Figure 2:**
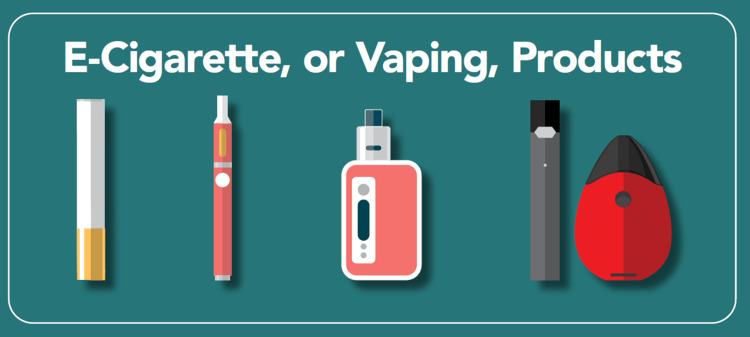
Types of e-cigarette devices. Source: US Centers for Disease Control and Prevention (public domain) [4].

E-cigarettes now encompass a diverse range of devices and delivery systems (Figure 2), each offering unique user experiences and potential health risks. As these products continue to evolve and gain traction among adolescents, a clearer understanding of their health implications is essential.

This narrative review explores the risk factors and health consequences associated with adolescent e-cigarette use and examines current strategies for cessation.

Background

**Figure 3:**
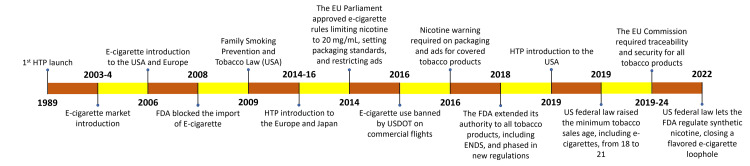
Historical timeline of electronic nicotine delivery systems (ENDSs) and regulatory policies. HTPs, Heated Tobacco Products; FDA, U.S. Food and Drug Administration; EU, European Union; USDOT, U.S. Department of Transportation Created using data from [1,2,5,10,19-25]

In 2003, Hon Lik, a Chinese pharmacist, introduced the current e-cigarette format [10]. Following the death of his father due to the long-term effects of smoking, Lik sought to develop a device that provided a safer method of nicotine delivery [10]. Since their introduction in the United States in 2006-7, e-cigarettes' popularity has been skyrocketing [2,5,6,8,11]. Due to health concerns and the FDA's classification of e-cigarettes as both a drug and a device, the agency intervened and banned all e-cigarette imports into the United States pending further approval (Figure 3) [1,2,5,10,19-25]. This did not last long, however, due to the Supreme Court siding with the nicotine industry [10]. In 2016, the FDA passed the Deeming Rule, which includes the prohibition of sales to minors, mandatory warning labels, and the requirement to undergo the FDA’s pre-market review and approval process [10].

## Review

Epidemiology

**Figure 4:**
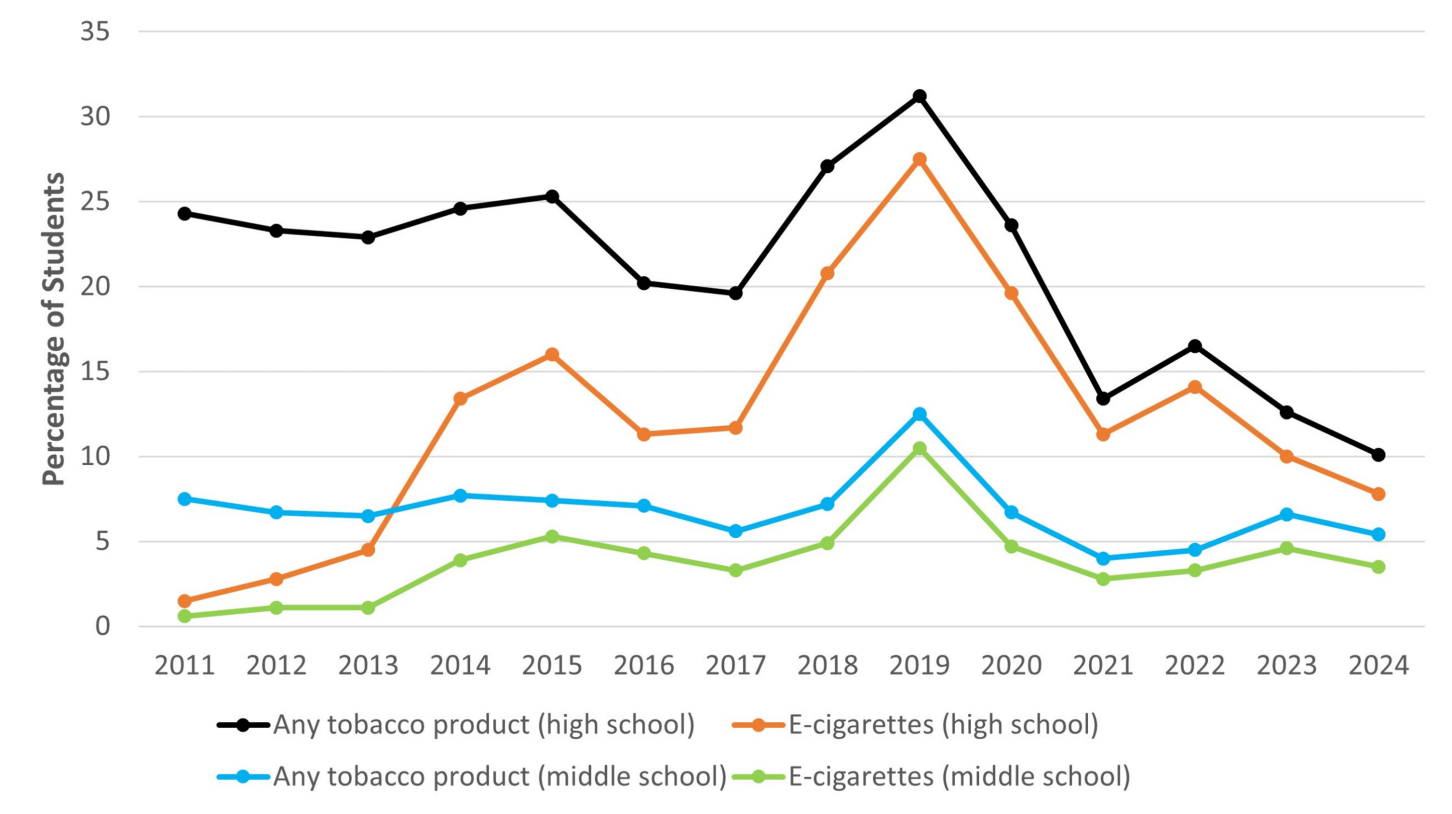
Percentage of middle and high school students who currently (past 30-day) use e-cigarettes and any tobacco product — National Youth Tobacco Survey, United States, 2011–2024. Created using data from [8-14]

About 10% of U.S. high school students and 5% of middle school students reported using tobacco products in 2024 [12]. According to the United States National Youth Tobacco Survey, the percentage of middle and high school students who currently (in the past 30 days) use e-cigarettes and any tobacco product has been slowly decreasing in recent years (Figure 4) [12-18]. E-cigarettes are the most commonly used product among students and adolescents [5,6,12]. The prevalence of e-cigarette use is significantly higher among youth and among those with lower family income and white non-Hispanics [6]. A recent meta-analysis reported that the global prevalence of ever and current e-cigarette use among adolescents was approximately 17% and 5%, respectively [26]. Another meta-analysis found that the global prevalence of ever and current use of an electronic nicotine delivery system (ENDS) among school and college students was approximately 22% and 10%, respectively [27]. Although some studies suggest that global e-cigarette use among adolescents may be slightly more common in boys, other findings indicate no significant sex difference [27-31].

Regulation and policy

With the exponential rise of e-cigarettes since their introduction into the market, the FDA and other national organizations had to intervene [1,2,5,10,19-25]. After e-cigarettes were introduced to the United States in 2007 (Figure 3), the FDA intervened in 2008 and blocked the company 'Smoking Everywhere' from importing them into the country [6,21,25,32]. The following year, ‘Smoking Everywhere’ decided to take the decision to court, where the company's appeal was turned down [6,21,25,32]. The FDA gained even more power during the same year of 2009 as President Obama signed the Family Smoking Prevention and Tobacco Act into law [10,33]. In a recent ruling, the United States Supreme Court upheld the FDA’s authority to issue marketing denial orders (MDOs) for certain flavored e-cigarette products, overturning a previous decision by the United States Court of Appeals for the Fifth Circuit that had struck down the MDOs [34]. The FDA oversees all aspects of e-cigarettes, including their manufacturing, packaging, importation, labeling, advertising, sale, distribution, and promotion [1,25]. Under FDA regulations, individuals under the age of 21 are prohibited from purchasing e-cigarettes, whether in stores or online [1,25]. The European Union and other countries have varying levels of regulation regarding e-cigarettes (Figure 3).

Reasons for the popularity of e-cigarette use among adolescents

Similar to the smoking epidemics of the 20th century, the use of e-cigarettes among adolescents may be driven by social factors such as peer influence and the desire to appear popular or "cool" in school settings [9,31,35]. Additional motivations include curiosity, appealing flavors and aromas, perceived health benefits, attempts to quit traditional smoking, and the influence of family members who use e-cigarettes [9,35,36]. Other contributing factors include the relatively low cost, widespread availability, discreet design, and overall convenience-particularly the ease with which e-cigarettes can be hidden and used in school environments [9,35-38].

Interviews with adolescents found that the first trial of an e-cigarette occurred due in part to curiosity, that it was welcomed by their peers, and could be used to cope with stress and belong to a group [37]. Furthermore, adolescents reported being more open to opportunities to vape once they found themselves in spaces away from authority figures and where declining an offer to vape would be socially difficult [37]. Lastly, despite potential concerns about health or nicotine dependence, adolescents believe that vaping provided a sense of belonging within a peer group and a way to alleviate stress and anxiety [37].

Additionally, experiences and age significantly affect usage of e-cigarettes versus combustible cigarettes, with e-cigarette users skewing younger and combustible cigarette users tending to be older [29]. Gender differences show that women are less likely to use combustible or e-cigarettes than men, and racial disparities appear, with Black or African American individuals significantly less likely to use e-cigarettes [29]. Higher education and income are associated with lower usage of both combustible and e-cigarettes, with the lowest odds among those with advanced degrees, while lower levels of mental health increase the likelihood of using combustible and e-cigarette products [29]. 

Toxicology

Given the wide variety of delivery systems and chemical components in e-cigarettes, there is significant heterogeneity among products, making it difficult to draw robust conclusions [10,39]. The major components of e-cigarettes include flavorings, nicotine, humectants, thermal degradation products, and metals [2,10,39]. More than 7,000 unique e-liquid flavors are available on the market, most of which have been tested for ingestion rather than inhalation [10,39]. The short- and long-term health effects of these chemicals, especially when aerosolized, remain inadequately studied and their safety is not well established [2,10,25,39].

Respiratory Toxicity

Growing evidence points to the respiratory effects of e-cigarettes, which may stem from nicotine exposure or other toxic substances. The oral cavity is often the first system affected, with studies suggesting alterations in the oral microbiome, increased inflammation in periodontal and peri-implant tissues, heightened susceptibility to dental caries, and potential cellular changes that could elevate the risk of oral premalignancy or malignancy [10,39,40]. However, a recent meta-analysis suggests that traditional combustible cigarettes have a more severe impact on periodontal tissues than e-cigarettes [41]. Nasal toxicity caused by exhaled e-cigarette aerosols has been associated with a decrease in immune-related gene expression [10]. The lower respiratory tract can be affected by e-cigarette use both acutely and chronically. The 2019 outbreak of E-cigarette or Vaping Use-Associated Lung Injury (EVALI), which resulted in approximately 3,000 hospitalizations, underscored the serious health risks associated with these products [10,42,43]. In addition, e-cigarette use may contribute to the development or worsening of chronic lung diseases such as asthma and chronic obstructive pulmonary disease [10,43-45].

Cardiovascular Toxicity

The effects of traditional combustible cigarette smoking on the cardiovascular system are well-documented [2,10]. A recent meta-analysis found that the primary effects of e-cigarette use include an increased heart rate and elevated blood pressure [46]. However, the cardiovascular impact of e-cigarette use remains insufficiently studied [10,43,47]. Emerging evidence suggests a potential association between e-cigarette use and adverse outcomes such as chest pain, coronary artery disease, and arrhythmias [10,43,47].

Health Effects on Adolescents

The impact of nicotine on the developing adolescent brain is well documented. It can affect brain circuits responsible for attention and learning, leading to mood disorders and impaired impulse control [9,25]. Nicotine also influences the brain’s reward system, increasing the risk of addiction and potentially enhancing the pleasurable effects of other substances, such as cocaine and methamphetamine [9,25].

The long-term consequences of nicotine exposure during adolescence remain largely unknown. However, recent predictive analyses concerning the global prevalence of smoking present alarming trends. While these projections are not specific to e-cigarette use, the associated risks of nicotine exposure may reasonably be inferred [48]. According to the study, the global age-standardized smoking prevalence in 2022 was estimated at approximately 29% among men and 6% among women [48]. Under their reference scenario, the authors projected a cumulative loss of about 29 billion years of life among men and 22 billion years among women between 2022 and 2050 [48].

Passive Exposure

Although studies on the effects of secondhand e-cigarette or vaping exposure are limited, existing data from traditional cigarette smoking strongly highlight the risks associated with nicotine and other chemical exposures [2,49]. One study found that switching from smoking to vaping indoors may significantly reduce, but not eliminate, children's secondhand exposure to nicotine and other harmful substances [50].

Addiction and dependency

Nicotine, a primary ingredient in e-cigarettes, can lead to addiction and dependency, as neuroadaptations can occur with the persistent use of nicotine (e.g., tolerance), withdrawal symptoms can be experienced when intake of nicotine is stopped, and the effects of nicotine that reinforce dependence [51]. The addictiveness of e-cigarettes has been found to be similar to cigarettes in terms of level of dependence and prevalence [52]. Additionally, levels of e-cigarette dependence were positively associated with nicotine concentration in e-liquid, as well as frequency and duration of e-cigarette use [52]. Disposable vapes show they deliver nicotine just as rapidly, and sometimes more intensely, than cigarettes, with fast absorption increasing satisfaction and reinforcing usage, especially among young people, thus increasing risk for addiction and dependency [53].

Connection to other substances and drugs

E-cigarette use is strongly tied to other substance use that can harm young adults and make nicotine cessation more difficult [2]. For example, nicotine use patterns were strongly associated with greater likelihood of cannabis use and binge drinking, particularly for the highest levels of each [54]. Those who smoked and vaped nicotine had about 37 times higher odds of having 10+ binge drinking instances in the past two weeks compared to non-users of nicotine [54].

E-cigarette use (vaping) cessation

E-cigarettes have been considered as possible tools to help people quit smoking. A systematic review has indicated that there is high-certainty evidence that e-cigarettes with nicotine increase quit rates compared to nicotine replacement therapy and moderate-certainty evidence that they increase quit rates compared to e-cigarettes without nicotine [55,56]. In a systematic review of nine studies, the authors concluded that individuals who switched from traditional smoking to e-cigarette use may experience a consistent pattern of weight gain over time [57]. However, to date, the FDA has not approved e-cigarettes to help people quit smoking.

## Conclusions

E-cigarette use among adolescents has been on the rise over the past two decades. The availability of flavored products and marketing that promotes vaping as a safer alternative to traditional combustible cigarettes have been major drivers of this trend. With scientific evidence on the long-term risks still emerging, education initiatives and advocacy for stronger legislation are essential. Given the lack of strict oversight on the contents of these products, regulatory bodies like the FDA must implement more rigorous controls to protect this vulnerable population. We must learn from the history of the tobacco industry and the well-documented short- and long-term health consequences it has caused.
